# Exercise, Free Radical Metabolism, and Aging: Cellular and Molecular Processes

**DOI:** 10.1155/2016/3813680

**Published:** 2016-02-02

**Authors:** Geraint D. Florida-James, Rickie Simpson, Gareth Davison, Graeme Close

**Affiliations:** ^1^School of Life Sport and Social Sciences, Edinburgh Napier University, Edinburgh EH11 4BN, UK; ^2^Laboratory of Integrated Physiology, University of Houston, 3855 Holman Street, Room 104 Garrison, Houston, TX 77204-6015, USA; ^3^Sport and Exercise Science Research Institute, Ulster University, Jordanstown, Newtownabbey BT37 OQB, UK; ^4^School of Sport and Exercise Sciences, Liverpool John Moores, Tom Reilly Building, Byrom Street, Liverpool L3 3AF, UK

The human aging process is associated with a gradual and cumulative decline in the normal functioning of all major bodily systems. While life expectancy is increasing at an exponential rate, the length of time spent in good health across the lifespan (health-span) is in decline. Exercise and physical activity are widely regarded as important interventions to increase longevity and promote healthy aging and well-being. However, cell damage produced by acute and unaccustomed exercise, as a result of an enhanced production of free radical species, is a recognised phenomenon. While all physiological systems appear responsive to the beneficial effects of exercise, further research is required to understand the relationship between free radical metabolism in cellular and molecular processes affected by exercise in the context of human aging. This special issue contains both review and original research articles which, when combined together, provide an interesting insight into the current state of basic and applied research in free radical metabolism, exercise, and aging.

In their review, O. F. Araneda et al. discuss how environmental factors, such as altitude and pollution, as well as different forms of exercise, intensity, and duration, affect pulmonary inflammation and oxidative imbalance. They review literature pertaining to antioxidants as well as prooxidants, in relation to oxidative damage, and they further discuss mediators of the inflammatory response to exercise. Importantly, they investigate the relationship between oxidative/inflammatory variables and the interaction between them.

D. J. Flis et al. used a prolonged swimming model in rats to investigate mitochondrial swelling and changes in cholesterol in skeletal muscle and liver. A reduction in mitochondrial cholesterol following exercise is related to the inhibition of mitochondrial swelling, and it was also observed within this study that there was an increase in caveolin-1 concentration in muscle. Although markers of oxidative stress in mitochondria were enhanced, there were no similar changes apparent in liver. The work points to a possible adaptive role of caveolin-1 in rat muscle mitochondria, as a consequence of exercise stress.

In the second review of this issue, M. Ross et al. discuss the increased risk of developing cardiovascular disease concurrent with increasing age, with respect to increased and prolonged exposure to oxidative stress. The positive and protective effect of regular exercise, in promoting endothelial homeostasis, which may offset the “vascular ageing” process, is further reviewed.

It is suggested that acute strenuous exercise causes changes to the oxidant/antioxidant ratio and tissue inflammation and, in their contribution to this issue, H. Li et al. add to the literature in this area with their report on the acute effect of exercise and the inflammatory response in the myocardium of rats. The rats were exercised using a treadmill running protocol, and they observed a regulatory adaptation of mitochondrial function in rat myocardium in response to acute heavy exercise. Their work further demonstrates that myocardial injury in rats is minimized via a cascade activation of mitophagy, responding to myocardial inflammation, as a result of mitochondrial stress triggered during acute exercise.

In the final research article of the issue, A. B. Bigley et al. focus on age, with regard to the effect of latent cytomegalovirus on natural killer cell phenotype and exercise responsiveness in the human model. In response to an acute bout of exercise, they show an increased mobilization of NK-cells, which is thought to be an important component of the “fight or flight” response. They also observed that this response is mediated by age, in reporting an increased proportion of NK-cells expressing the terminal differentiation marker CD57 with advancing years. Additionally, NK-cell mediated immunosurveillance following exercise was reported to be compromised by CMC serostatus irrespective of age.

Although there is certainly great diversity in the papers presented in this special issue, we hope the reader will find the contributions both interesting and stimulating. We suggest that this collection of papers will serve to enrich our understanding and knowledge of the relationship between free radical metabolism in cellular and molecular processes affected by exercise in the context of aging.

*Geraint D. Florida-James* *Geraint D. Florida-James* *Rickie Simpson* *Rickie Simpson* *Gareth Davison* *Gareth Davison* *Graeme Close* *Graeme Close*

